# Identification of genes associated with histologic tumor grade of esophageal squamous cell carcinoma

**DOI:** 10.1002/2211-5463.12228

**Published:** 2017-07-28

**Authors:** Jiaqiang Xing, Cuicui Liu

**Affiliations:** ^1^ Department of Thoracic Surgery Linyi Cancer Hospital of Shandong Province China; ^2^ Department of Oncology The People's Hospital of Linyi Shandong China

**Keywords:** esophageal squamous cell carcinoma, expression profiling, histologic tumor grade, receiver operating characteristic, *The Cancer Genome Atlas*

## Abstract

The present study aimed to identify the genes associated with the histologic tumor grade of patients with esophageal squamous cell carcinoma (ESCC) and to provide valuable information for the identification of potential diagnostic biomarkers in ESCC. Tumor samples of ESCC patients retrieved from *The Cancer Genome Atlas* were divided into Grade 1 (well‐differentiated; G1), Grade 2 (moderately‐differentiated; G2) and Grade 3 (poorly‐differentiated; G3) groups in accordance with the clinical record of the tumor grade of ESCC patients. The genes associated with tumor grade were identified. The signaling pathways of identified genes were enriched from the *Kyoto Encyclopedia of Genes and Genomes* (KEGG). The diagnostic value of candidate genes was assessed by receiver operating characteristic analysis. We used the GSE23400 dataset generated from the Gene Expression Omnibus to examine the expression levels of candidate genes in ESCC tissues compared to matched mucosa tissues. In total, 440 genes positively correlated with tumor grade and 882 genes negatively correlated with tumor grade were identified. There were 310 differentially expressed genes (DEGs) between G1 and G2, 184 DEGs between G2 and G3, and 710 DEGs between G1 and G3. There were 1322 genes associated with tumor grade that were significantly enriched in pathways in cancer and the phospholipase D signaling pathway. Cyclin‐dependent kinase inhibitor 1A, golgin A7 family member B and transforming growth factor B1‐induced anti‐apoptotic factor 1 (*TIAF1*) had potential diagnostic value for discriminating ESCC patients with G1 from those with G3. *TIAF1* was significantly down‐regulated in ESCC. The results of the present study comprise useful groundwork with respect to determining the tumorigenesis mechanism in ESCC and discovering potential diagnostic biomarkers for ESCC.

**Abbreviations**
ARNT2: aryl hydrocarbon receptor nuclear translocator 2
AUC: area under the curve
BCR: biospecimen core resource
BDKRB2: bradykinin receptor B2
BE: Barrett's esophagus
CA12: carbonic anhydrase 12
CABC1: coenzyme Q8A
CDKN1A: cyclin‐dependent kinase inhibitor 1A
DEG: differentially expressed gene
EAC: esophageal adenocarcinoma
EC: esophageal carcinoma
ESCC: esophageal squamous cell carcinoma
GEO: Gene Expression Omnibus
GERD: gastroesophageal reflux disease
GNA15: G protein subunit α15
GOLG: golgin
HCC: hepatocellular carcinoma
JUP: junction plakoglobin
KEGG: *Kyoto Encyclopedia of Genes and Genomes*
LAD1: ladinin 1
LAMB3: laminin subunit β3
NSCLC: nonsmall cell lung cancer
PHLPP: PH domain and leucine rich repeat protein phosphatase
PKP1: plakophilin 1
PPAR: peroxisome proliferator activated receptor
PRSS8: protease, serine 8
ROC: receiver operating characteristic
TCGA: *The Cancer Genome Atlas*
TIAF1: transforming growth factor B1‐induced anti‐apoptotic factor 1
TMEM108: transmembrane protein 108
TNM: tumor, node and metastasis
TSS: tissue source site

Esophageal carcinoma (EC) is a common malignancy of digestive tract carcinoma worldwide, with an estimated 455 000 new cases and 400 000 deaths each year 1. EC is classified as esophageal squamous cell carcinoma (ESCC) and esophageal adenocarcinoma (EAC) in accordance with the histopathological appearance of tumor tissues.

Smoking, a low intake of vegetables and fruits, being overweight, obesity, chronic gastroesophageal reflux disease (GERD) and Barrett's esophagus (BE) are risk factors in EAC 1, 2. In western countries, the incidence rate of GERD is almost 18% 3. BE, with the phenotypic change of normal esophageal squamous epithelium to a columnar and intestinal‐type epithelium, is associated with an increased risk of EAC 2. A meta‐analysis indicated that the morbidity of ESCC cases with human papillomavirus infection is highest in Asia and Africa, especially in China 4. EAC is the dominant subtype in European countries, whereas ESCC accounts for 90% of EC cases in Asian countries, such as China and Iran 4.

The histologic tumor grade of ESCC is segregated into Grade 1 (well‐differentiated), Grade 2 (moderately‐differentiated) and Grade 3 (poorly‐differentiated) according into the World Health Organization classification. Tumor grade is an independent prognostic factor of EC 5. ESCC patients with poor differentiation, have a poorer prognosis compared to those with well‐ and moderate differentiation.

It is acknowledged that the intersection of etiological factors, including dysregulated genes, aberrant expression of micorRNAs and abnormal DNA methylation, contributes to the initiation and development of ESCC. Over‐expression of miR‐483‐3p results in a poor prognosis for patients through promoting ESCC progression as a result of targeting EI24 6. An increased expression level of miR‐224 is associated with advanced tumor, node and metastasis (TNM) stage and tumor grade, which promotes cell proliferation, migration, invasion and suppresses cell apoptosis of ESCC cells by targeting PH domain and leucine rich repeat protein phosphatase (PHLPP)1 and *PHLPP2* 7. The expression of protease, serine 8 (*PRSS8*) with hypermethylation is significantly decreased in ESCC, which predicts a shorter overall survival of patients with ESCC. Demethylation of *PRSS8* contributes to the inhibition of tumor progression, including cell proliferation, migration and cell cycle arrest 8. However, the tumorigenesis mechanism of ESCC is unclear.

In the present study, bioinformatics analyses were performed to identify the dysregulated genes and pathways correlated with histologic tumor grade based on the expression profiling of ESCC in *The Cancer Genome Atlas* (TCGA) database. We aimed to provide the groundwork with respect to determining the tumorigenesis mechanism in ESCC, as well as discovering potential diagnostic biomarkers.

## Materials and methods

### Sample collection

The present study used sequence‐related data from ESCC tissues from the TCGA database. ESCC tissues and clinical metadata of eligible ESCC patients were collected by Tissue Source Sites (TSSs), such as the University of Alabama, the Technical University of Munich and the University of Kansas Medical Center. After a preliminary pathological review, TSSs deliver ESCC tissue samples and metadata to the Biospecimen Core Resource (BCR). Next, the BCR verifies the quality and quantity of the pathological diagnosis of ESCC tissues. The RNA is then extracted from ESCC tissues and also by BCR for genomic characterization and high‐throughput sequencing. Sequence‐related data are deposited in the TCGA database 9.

### Basic information of esophageal squamous cell carcinoma patients

A total of 188 esophageal squamous cell carcinoma patients with clinical records (collected from 26 June 2012 to 28 January 2015) were available in the TCGA database. The tumor grade of ESCC samples is recorded, which was divided into five grade groups, such as GX (unknown), G1 (well‐differentiated), G2 (moderately‐differentiated), G3 (poorly‐differentiated) and G4 (undifferentiated), in accordance with the World Health Organization classification.

The inclusion criteria of patients were patients: (a) with a subtype of esophageal squamous cell carcinoma; (b) without a history of other malignancy; (c) without a history of neoadjuvant treatment; (d) for whom the expression profiling of mRNA was available; and (e) for whom the record of histologic tumor grade was G1–G3. In the present study, ESCC patients were separated into G1, G2 and G3 groups in accordance with the recorded tumor grade. Level 3 mRNA sequence data of ESCC patients were downloaded from the TCGA data portal, which is based on UNC Illumina Hiseq_RNASeqV2.

### The correlation of the expression of mRNAs with tumor grade

Those mRNAs with a 0 reads count were excluded from the study. A linear by linear association test 10 was applied to analyze the correlation of the expression of genes with tumor grade by using the lbl.test function of the coin package in r 11. *P* < 0.05 was considered statistically significant.

### Box‐plot analyses and hierarchical clustering analyses

The significant correlations between expression levels of genes and tumor grade were visualized via a Box‐plot analysis in r 12. Two‐way hierarchical clustering analyses were applied to assess the similarity of gene expression patterns among G1, G2 and G3 groups, and were visualized via the ‘pheatmap’ package in r 13.

### Identification of differentially expressed genes

The genes associated with tumor grade were identified. To clarify the expression specificity of those genes, the significance analyses of differentially expressed genes between G1 and G2 groups, between G2 and G3 groups, and between G3 and G3 groups were subjected to Tukey's honest significant difference 14. *P* < 0.05 was considered statistically significant.

### *Kyoto Encyclopedia of Genes and Genomes* (KEGG) pathway enrichment

To obtain insights into the signaling pathways of genes associated with the tumor grade of ESCC, KEGG pathway enrichment was performed using genecodis3 15, 16. *P* < 0.05 was considered statistically significant.

### Receiver operating characteristic (ROC) curve analysis

To assess the diagnostic value of candidate genes in ESCC (G1 and G3 groups), the ROC curve was deciphered and the area under the curve (AUC) was calculated to assess the diagnostic value of aryl hydrocarbon receptor nuclear translocator 2 (*ARNT2*), *GOLGA7B*, transforming growth factor B1‐induced anti‐apoptotic factor 1 (*TIAF1*), cyclin‐dependent kinase inhibitor 1A (*CDKN1A*), peroxisome proliferator activated receptor (PPAR)G and laminin subunit β3 (*LAMB3*) in ESCC via the pROC package in r.

### The expression levels of candidate genes were validated based on the Gene Expression Omnibus (GEO) database

The expression levels of candidate genes in ESCC tissues compared with adjacent associated nontumor tissues was not validated based on the mRNA sequence date generated from the TCGA database because only two ESCC adjacent nontumor mucosa samples were available in the TCGA database.

To examine the expression levels of candidate genes in ESCC tissues, the microarray expression profiling of ESCC tissues and adjacent nontumor tissues were obtained from GEO database (https://www.ncbi.nlm.nih.gov/geo/), which is a freely public data repository that archives microarray, next‐generation sequencing and other forms of high‐throughput functional genomics data submitted by the research community.

The microarray expression profiling of ESCC issues was searched and the inclusion criteria of datasets required that: (a) the dataset was generated from the mRNA expression profiling of ESCC patients; (b) both the ESCC and adjacent nonumor mucosa tissues samples were available in the dataset; (c) the sample size of dataset was greater than 50; and (d) sequence data of the candidate genes were available in the dataset. Finally, GSE23400 (53 ESCC versus 53 adjacent nontumor mucosa tissues) were incorporated in the present study.

Box‐plot analyses were performed to describe the expression of candidate gene both in ESCC and matched mucosa tissues. The *P*‐value indicating the difference between two group was calculated. *P* < 0.05 was considered statistically significant.

## Results

### Basic information

A total of 83 ESCC patients were incorporated into the present study based on the exclusion criteria. In total, 16, 47 and 20 patients with ESCC were assigned to the G1, G2 and G3 groups, respectively. There were 20 531 genes available in the mRNA sequence data of the ESCC tumor based on the Illumina Hiseq_RNASeqV2 in TCGA database. After trimming the 6026 genes with a 0 reads count, 14 506 genes were included in the present study.

### Identification of genes associated with the histological grade of ESCC

**Figure 1:**
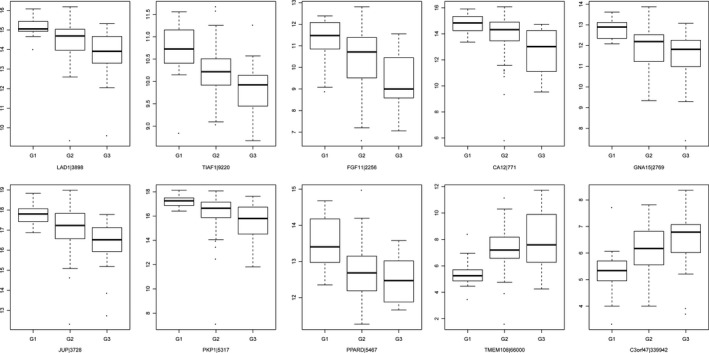
Heat map visualization of the expression pattern of the top 100 genes associated with the tumor grade of ESCC in the G1, G2 and G3 groups. Red represents up‐regulation and green represents down‐regulation.

Correlations between the expression of genes and tumor grade of ESCC patients were analyzed by a linear by linear association test. Eventually, 1322 genes associated with tumor grade were identified, including 440 genes positively correlated with tumor grade and 882 genes negatively correlated with tumor grade (Table S1). As shown in Table 1, transmembrane protein 108 (*TMEM108*), *C3orf47* and coenzyme Q8A (*CABC1*) were the top three genes positively correlated with tumor grade, whereas ladinin 1 (*LAD1*), *TIAF1* and *FGF11* were the top three genes negatively correlated with the tumor grade of ESCC patients. The respective expression status of genes in the G1, G2 and G3 groups is shown in Fig. 1. *TMEM108* and *C3orf47* were positively correlated with tumor grade and eight genes, including *LAD1*,* TIF1*,* FGF11*, carbonic anhydrase 12 (*CA12*), G protein subunit α15 (*GNA15*), junction plakoglobin (*JUP*), plakophilin 1 (*PKP1*) and *PPARD*, were negatively correlated with the tumor grade of ESCC patients.

**Table 1:** The genes associated with the histological grade of ESCC

Gene ID	Gene symbol	Mean G1	Mean G2	Mean G3	*P*‐value
Positive correlation					
66000	TMEM108	5.418252604	7.329616234	7.916632334	7.32E‐05
339942	C3orf47	5.267250937	6.198372806	6.497636229	0.0001191
56997	CABC1	9.73904574	10.85150959	10.80083313	0.0002155
56994	CHPT1	8.875674851	10.26804519	10.40381399	0.0002155
777	CACNA1E	1.946473596	3.895276537	4.321101557	0.0002574
2781	GNAZ	5.840673299	8.034275975	8.201352094	0.0002658
9915	ARNT2	7.913558745	9.449765716	9.896500029	0.000382
84446	BRSK1	5.704577382	7.004365547	7.552181962	0.000382
404217	CTXN1	7.802343062	8.209738979	9.276647634	0.000382
7275	TUB	6.942555084	8.447990872	9.069991495	0.000382
Negative correlation					
3898	LAD1	15.12740154	14.41469	13.78649	1.83E‐05
9220	TIAF1	10.70008046	10.29157	9.869611	3.53E‐05
2256	FGF11	11.26322247	10.4069	9.425334	4.05E‐05
771	CA12	14.77830133	13.78284	12.64873	6.64E‐05
2769	GNA15	12.81232253	11.95196	11.38613	6.64E‐05
3728	JUP	17.77287667	17.09011	16.28087	6.64E‐05
5317	PKP1	17.23345646	16.1469	15.54338	6.64E‐05
5467	PPARD	13.54200932	12.72773	12.43948	6.64E‐05
1830	DSG3	16.28279128	15.41625	14.01574	0.0001226
401647	GOLGA7B	10.61928628	9.106061	7.615288	0.0001226

G1, high differentiation; G2, middle differentiation; G3, low differentiation.

### KEGG pathway enrichment

In total, 1322 genes associated with the tumor grade of ESCC were significantly enriched in 14 signaling pathways, including pathways in cancer (hsa05200), the phospholipase D signaling pathway (hsa04072), small cell lung cancer (hsa05222) and gastric acid secretion (hsa04971) (Table 2).

**Table 2:** KEGG pathway enrichment

KEGG term	KEGG ID	Number	*P*‐value	Gene
Pathways in cancer	hsa05200	56	0.00057	*BDKRB1, GNAS, PIK3R3, BCL2, GNAI1, LAMA1, GNAI3, PLEKHG5, LAMA3, BIRC5, PLCB3, CXCL12, PLCB4, PLCG1, TPM3, ADCY7, ADCY6, ITGA6, LPAR1, ITGA3, GNB3, ARNT2, SLC2A1, DAPK1, GNG7, BDKRB2, RB1, RALGDS, TRAF5, CSF3R, STK36, WNT6, EGLN3, LEF1, CDH1, LAMB3, LAMC2, E2F3, COL4A4, JUP, TGFB1, PPARD, AXIN2, RALA, PPARG, LPAR5, GSTP1, CDKN1A, WNT4, VEGFA, RARB, FGF11, RELA, PGF, RXRA, FZD2*
Phospholipase D signaling pathway	hsa04072	24	0.00312	*GNAS, PIK3R3, SYK, PDGFC, SHC3, PLCB3, PLCB4, PLCG1, ADCY7, ADCY6, PLD2, DGKD, AGPAT4, LPAR1, RALGDS, PIP5K1B, LPAR5, RRAS2, ARF6, RALA, SPHK1, SHC1, DGKA, AGPAT2*
Small cell lung cancer	hsa05222	15	0.01286	*PIK3R3, COL4A4, BCL2, LAMA1, LAMA3, LAMC2, TRAF5, RB1, LAMB3, E2F3, RARB, ITGA6, RELA, RXRA, ITGA3*
Bacterial invasion of epithelial cells	hsa05100	14	0.013	*CLTB, PIK3R3, CAV1, ARPC3, PXN, CAV2, CDH1, SHC3, SHC1, ELMO1, SEPT3, ARPC5L, SEPT9, SEPT6*
Rap1 signaling pathway	hsa04015	29	0.01462	*GNAS, PIK3R3, PDGFC, GNAI1, GNAI3, PARD6A, PLCB3, PLCB4, PLCG1, ADCY7, ADCY6, LPAR1, PRKD1, EPHA2, TIAM1, CTNND1, EFNA3, RALGDS, PARD6G, CALML3, VEGFA, CDH1, LPAR5, RALA, PGF, PRKD3, MAP2K3, VASP, FGF11*
Inflammatory mediator regulation of TRP channels	hsa04750	16	0.01735	*BDKRB1, L1RAP, PIK3R3, MAP2K3, BDKRB2, PLCB3, PLCB4, PLCG1, PPP1CA, ADCY7, ADCY6, CALML3, F2RL1, GNAS, CAMK2B, CAMK2D*
Gastric acid secretion	hsa04971	13	0.01878	*GNAS, GNAI1, CA2, GNAI3, SLC9A1, PLCB3, PLCB4, EZR, ADCY7, ADCY6, CALML3, CAMK2B, CAMK2D*
Arrhythmogenic right ventricular cardiomyopathy	hsa05412	13	0.01878	*DSP, LEF1, CACNB2, LMNA, ITGB4, CACNA2D2, ITGA9, DSC2, JUP, DMD, GJA1, ITGA6, ITGA3*
Inositol phosphate metabolism	hsa00562	12	0.02948	*IMPA2, IPPK, INPPL1, PIP5K1B, PLCD3, PIP5KL1, PLCB3, PLCB4, PLCG1, ISYNA1, INPP4B, PIKFYVE*
Endocytosis	hsa04144	32	0.03457	*LDLR, ADRB2, GRK2, PARD6A, SH3GL1, PLD2, HSPA1L, VPS4A, HSPA1A, CAV1, DNAJC6, RAB10, CAV2, EHD1, PARD6G, RAB11FIP5, CLTB, HGS, EHD2, EHD4, PIP5K1B, RAB8A, ARF6, TGFB1, ACAP3, VPS37C, ARPC3, PIP5KL1, CAPZA1, EPN1, ARPC5L, SNX3*
Fc gamma R‐mediated phagocytosis	hsa04666	14	0.03937	*PIK3R3, SYK, SPHK1, ARPC3, INPPL1, PIP5K1B, CFL1, ARF6, PLCG1, VASP, MARCKSL1, PLD2, ARPC5L, RPS6KB2*
Glucagon signaling pathway	hsa04922	15	0.03975	*PYGL, GNAS, LDHA, PHKG1, PGAM1, SLC2A1, PKM, GYS1, PLCB3, PLCB4, PDE3B, CALML3, PGAM4, CAMK2B, CAMK2D*
Regulation of actin cytoskeleton	hsa04810	27	0.04176	*BDKRB1, PIK3R3, PDGFC, MSN, PPP1CA, ITGA6, BAIAP2, ITGA3, ARPC3, TIAM1, ITGB4, BDKRB2, CFL1, EZ, PIKFYVE, ABI2, PIP5K1B, RRAS2, DIAPH1, CYFIP2, ITGA9, PAK6, SSH3, SLC9A1, PXN, FGF11, ARPC5L*
Adrenergic signaling in cardiomyocytes	hsa04261	20	0.04288	*GNAS, PIK3R3, GNAI3, ADRB2, BCL2, GNAI1, CAMK2D, CACNA2D2, CACNB2, ADCY6, SLC9A1, PLCB3, PLCB4, PPP1CA, ADCY7, PPP2R5C, CALML3, SCN5A, CAMK2B, TPM3*

### The expression pattern of genes associated with tumor grade in the G1, G2 and G3 groups

**Figure 2:**
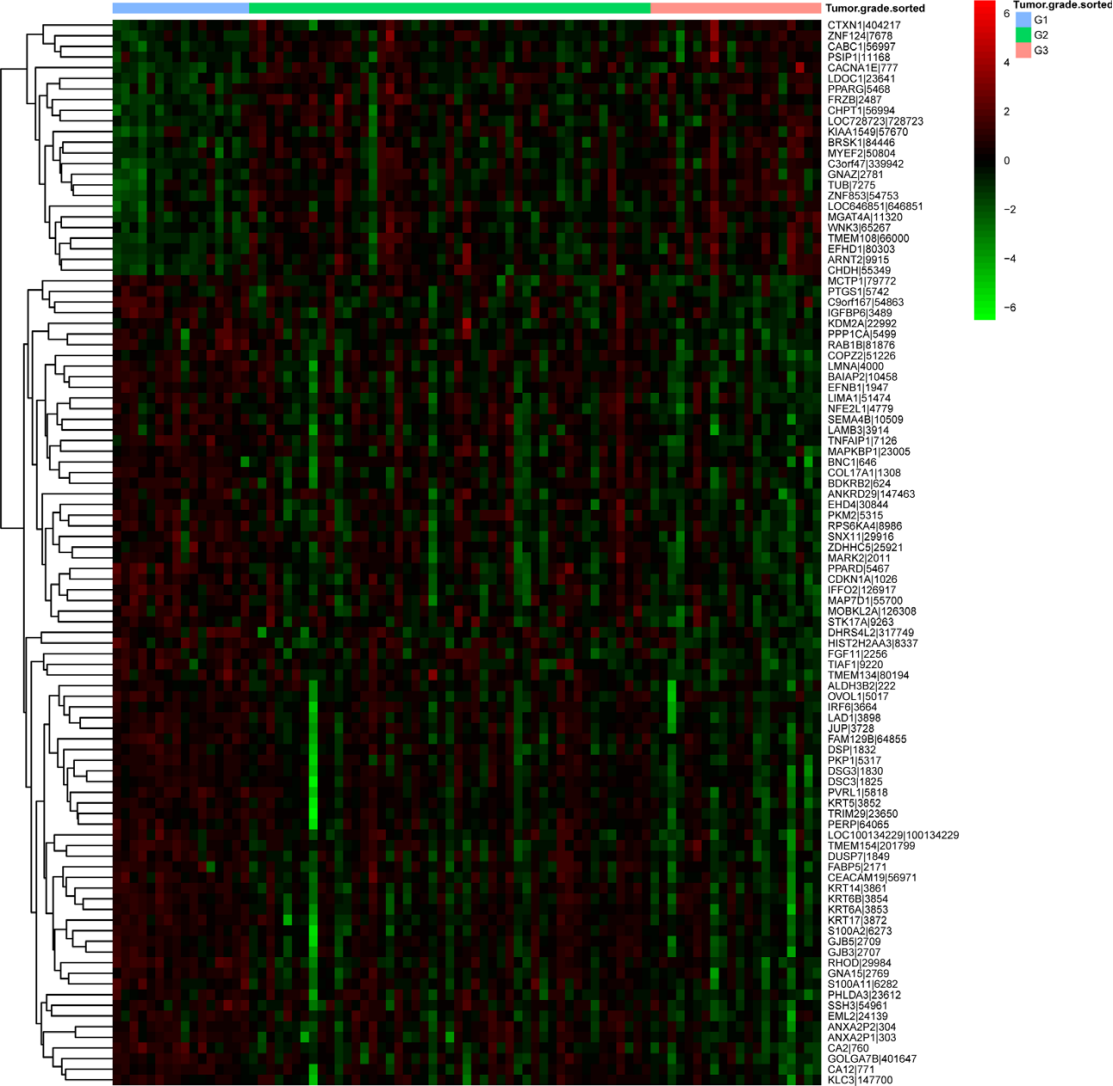
Box‐plot depicted the median and interquartile range of expression levels of candidate genes associated with the tumor grade of ESCC in the G1, G2 and G3 groups. The candidate genes were LAD1, TIAF1, FGF11, CA12, GNA15, JUP, PKP1, PPARD, TMEM108 and C3orf47.

To access the similarity of gene expression pattern among the G1, G2 and G3 groups, the top 100 genes associated with the tumor grade of ESCC were submitted to heatmap analyses. As shown in Fig. 2, an obvious difference in gene expression pattern was observed between the G1 and G2 groups, as well as between the G1 and G3 groups.

### Differentially expressed genes analysis

We identified 1322 genes associated with the tumor grade of ESCC, althoguh whether these genes were significantly differentially expressed among the G1, G2 and G3 groups requires further analyses.

**Figure 3:**
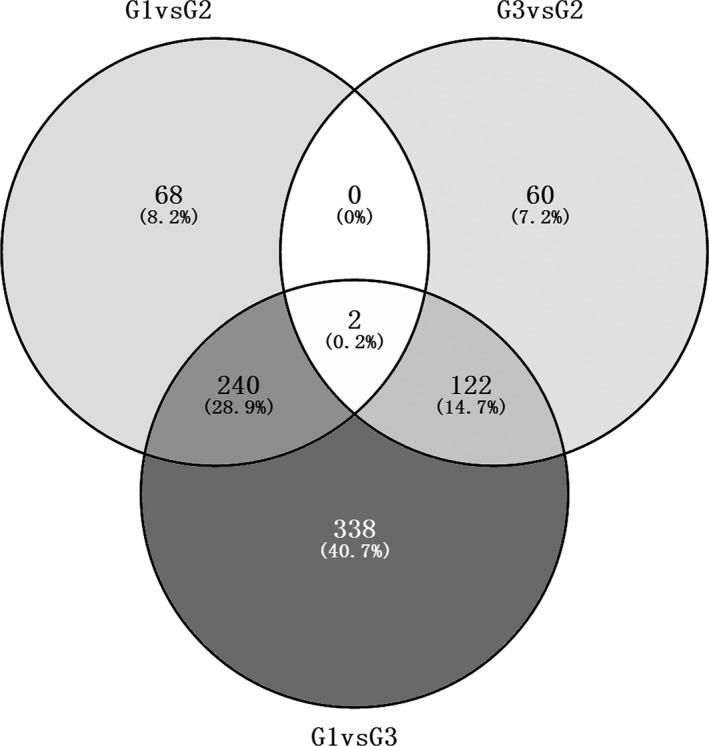
Venn diagram showing the overlap of differentially expressed genes correlated with the tumor grade of ESCC between the G1 and G2 groups, between the G2 and G3 groups, and between the G1 and G3 groups. G1, G2 and G3 indicate grade 1, grade 2 and grade 3 of ESCC, respectively. G1 versus G2 indicates differentially expressed genes between the G1 and G2 groups. G3 versus G2 indicates differentially expressed genes between the G2 and G3 groups. G1 versus G3 indicates differentially expressed genes between the G1 and G3 groups.

For 1322 genes, the significant difference of the expression level of each gene between the G1 and G2 groups (G1 versus G2), between the G2 and G3 groups (G2 versus G3) and between the G1 and G3 (G3 versus G1) groups was analyzed. Genes showing a significant difference (*P* < 0.05) were identified as being differentially expressed genes. There were 310, 184 and 710 differentially expressed genes, respectively, identified in G1 versus G2, G2 versus G3 and G3 versus G1 (Table S2). As shown in Fig. 3, two of genes were overlapped from 310, 184 and 710 differentially expressed genes, which were *GOLGA7B* and *TIAF1*.

### ROC curve analysis

**Figure 4:**
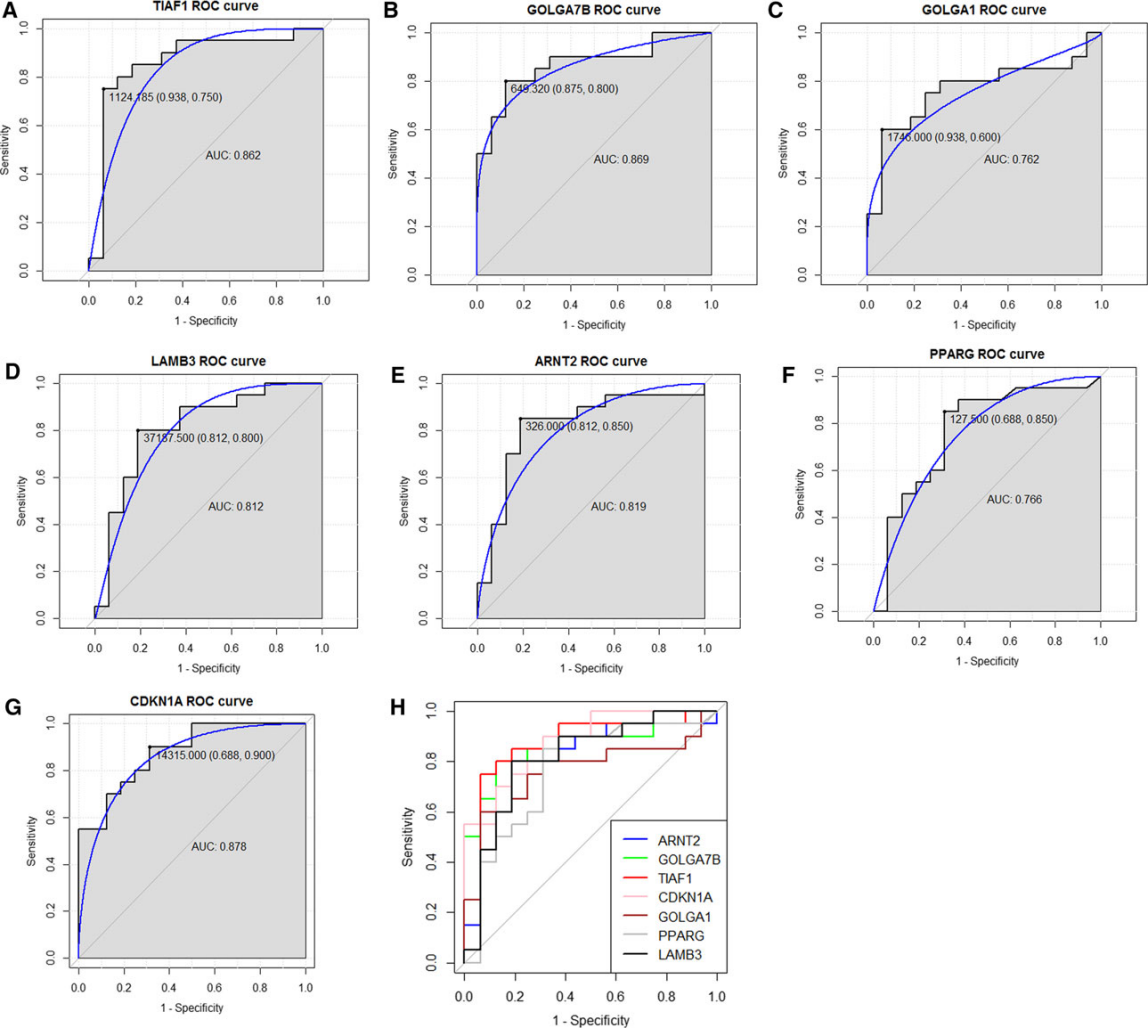
The discriminatory ability of the candidate genes between ESCC patients with G1 and those with G3 was accessed by ROC curve analyses. (A) ROC curve of TIAF1. (B) ROC curve of GOLGA7B. (C) ROC curve of GOLGA1. (D) ROC curve of LAMB3. (E) ROC curve of ARNT2. (F) ROC curve of PPARG. (G) ROC curve of CDKN1A. (H) The merged ROC curves of TIAF1, GOLGA7B, GOLGA1, LAMB3, ARNT2, PPARG and CDKN1A.

To identify the discriminatory ability of the seven candidate genes between ESCC patients with G1 and those with G3, ROC curves were depicted and the AUC was calculated. As shown in Fig. 4 shown, the AUC of *TIAF1* (0.862), *GOLGA7B* (0.869), *LAMB3* (0.812), *ARNT2* (0.819) and *CDKN1A* (0.878) was greater than 0.8; the AUC of *GOLGA1* (0.762) and *PPARG* (0.766) was less than 0.8. *CDKN1A*,* GOLGA7B* and *TIAF1* had a larger AUC than the other four candidate genes. The sensitivity and specificity of *CDKN1A* was 90% and 68.8% (Fig. 4G), the sensitivity and specificity of *GOLGA7B* was 80% and 87.5% (Fig. 4B) and the sensitivity and specificity of *TIAF1* was 75% and 93.8%, respectively (Fig. 4A).

### The expression levels of candidate genes were analyzed in the GSE23400 dataset

**Figure 5:**
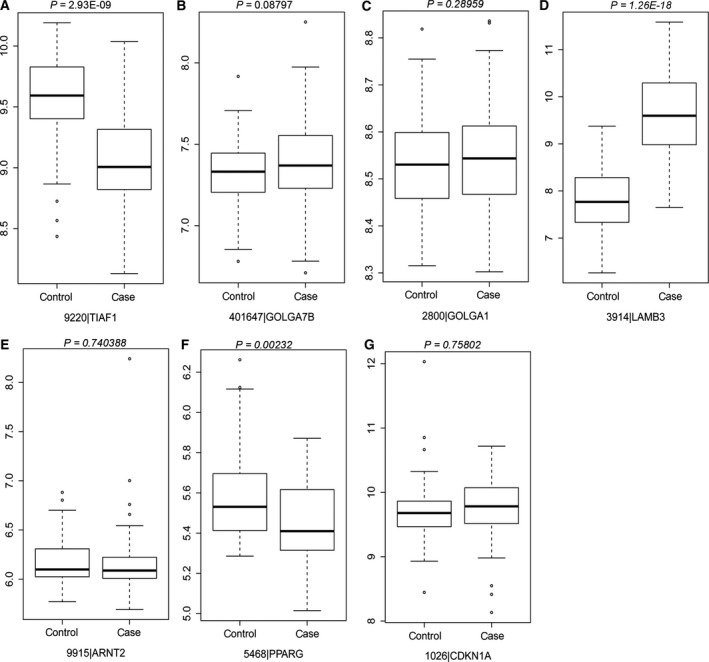
Box‐plot analyses presented the expression levels of seven candidate genes in ESCC tissues and matched mucosa tissues in the GSE23400 datasets. (A) *TIAF1*. (B) *GOLGA7B*. (C) *GOLGA1*. (D) *LAMB3*. (E) *ARNT2*. (F) *PPARG*. (G) *CDKN1A*. Case indicates ESCC tissues and normal indicates adjacent nontumor mucosa tissues.

The expression levels of seven candidate genes were detected in GSE23400 datasets, such as *TIAF1, GOLGA7B, GOLA1, LAMB3, ARNT2, PPARG* and *CDKN1A*. As shown in Fig. 5, the difference in expression levels of *GOLGA7B* (Fig. 5B), *GOLGA1* (Fig. 5C), *ARNT2* (Fig. 5E) and *CDKN1A* (Fig. 5G) between ESCC and matched mucosa tissues was not significant. The expression levels of *TIAF1* (Fig. 5A) and *PPARG* (Fig. 5F) were significantly down‐regulated in ESCC tissues and *LAMB3* (Fig. 5D) was significantly up‐regulated in ESCC tissues compared to matched mucosa tissues.

## Discussion

*TIAF1* locates at chromosome 17 and encodes TGFB1‐induced anti‐apoptotic factor 1. In our analyses, the expression level of *TIAF1* was negatively correlated with histological grade. It was significantly up‐regulated in G2 (moderately‐differentiated) compared to G3 (poorly‐differentiated) and significantly up‐regulated in G1 compared to G2. In the GSE23400 dataset, it was significantly down‐regulated in ESCC tissues compared to matched mucosa tissues. In addition, *TIAF1* could discriminate between ESCC patients with well‐differentiated ESCC from those with poorly‐differentiated ESCC. The results of the present study indicate that TIAF1 might function as a tumor suppressor in the development of ESCC and might also be a potential biomarker for ESCC diagnosis. TIAF1 contributes to the progress of Alzheimer's disease and tumor 17, 18, 19. Self‐aggregation of TIAF1in the human hippocampus leads to the generation of amyloid β plaques, which results in neurodegeneration in Alzheimer's disease 18. TIAF1 is the key component for tumor suppressors of p53 and WW domain containing oxidoreductase, which mediates tumor suppression and apoptosis and is frequently scarce in metastatic lung cancer cells 19. TIAF is up‐regulated in nonmetastatic prostate cancer and down‐regulated in breast cancer 17. In histiocytic lymphoma U937 cells, TIAF1 up‐regulates the expression of p53 and induces the inhibition of cell growth and cell apoptosis by suppressing ERK phosphorylation 20. The present study first reported a reduction of the higher expression of TIAF1 associated with well‐differentiated ESCC, and TIAF1 might function as a tumor suppressor in ESCC cell proliferation, invasion and metastasis. Further work is needed to explore the biological roles of TIAF1 in the development of ESCC and to translate the prognostic value of TIAF1 into clinical practice.

In the present study, the expression levels of *GOLGA7B* and *GOLGA1* in ESCC patients with G3 (poorly‐differentiated) were significantly lower than those with G2 (moderately‐differentiated) and G1 (well‐differentiated). *GOLGA7B* locates at chromosome 10 and encodes GOLGA7B. *GOLGA1* locates at chromosome 8 and encodes golgin A1. The exact roles of GOLGA7B and GOLGA1 remain unknown. GOLGAl is reported to be stably expressed in 17 tissues samples, including breast, esophagus, lung, pancreases, colon, rectum and duodenum 21. *GOLGA2* encodes golgin A, another member of golgin family, which is required for efficient tracking in the secretory pathway at the Golgi complex 22. *In vivo* mice model experiments show that down‐regulation of GOLGA2 suppresses lung cancer tumorigenesis by inhibiting angiogenesis and cell invasion 23. In addition, GOLGA2 associates with the susceptibility to galectin‐1‐induced cell apoptosis in advanced prostate cancer 24. *GOLGA7B* and *GOLGA1* were identified as novel genes associated with the tumor grade of ESCC. The biological roles of GOLGA7B and GOLGA1 in ESCC are unknown. Further investigation is necessary to clarify the mechanism of GOLGA7B and GOLGA1 with respect to the initiation and development of ESCC through *in vitro* and *in vivo* experiments.

A total of 56 genes associated with the tumor grade of ESCC was significantly enriched in pathways in cancer (Table 2). Eight key genes, including *ARNT2, PPARG, PPARD, FGF11, JUP, CDKN1A*, bradykinin receptor B2 (*BDKRB2*) and *LAMB3*, overlapped from the 56 genes mentioned above, and 702 differentially expressed genes between the G3 and G1 groups and the top 50 genes negatively/positively correlated with tumor grade were obtained.

*LAMB3* encodes laminin subunit α3 and belongs to laminin family of extracellular matrix glycoproteins (secreted molecules) that have a heterotrimeric structure consisting of α, β and γ subunits. *LAMB3*, along with *LAMA1*,* LAMA3* and *LAMC2*, was enriched in pathways involved in cancer. Figure 5D shows that LAMB3 was significantly up‐regulated in ESCC tissues compared to matched mucosa tissues, for which the results are not compatible with our bioinformatics analyses, indicating that *LAMBA3* was significantly down‐regulated in ESCC with G3 (poorly‐differentiated) compared to those with G1 (well‐differentiated) and G2 (moderately‐differentiated). However, the expression status of LAMB3 in GSE23400 was in agreement with the previous studies. The expression level of LAMB3 is reported to be up‐regulated in ESCC compared to normal tissues and LABM3 expression is correlated with the depth of invasion and malignancy 25. In addition, LAMB3 functions as an oncogene in cervical squamous cell carcinoma 26. LAMB3, LAMA3 and LAMC2 constitute lamin‐5, which connects epithelium cells to the underlying basement membrane and regulates cell migration, adhesion and mechanical signal transduction 27. Laminin‐5 is up‐regulated in ESCC and its expression is associated with TNM stage and a poor prognosis for patients. Laminin 5 promotes ESCC cell invasion by activating the phosphoinositide 3‐kinase pathway 28. Accordingly, the laminin family might play a vital role in ESCC cell differentiation, adhesion and migration. The functions of LAMB3 as an oncogene or a tumor suppressor in ESCC carcinogenesis need to be explored in further work.

*ARNT2* and *PPARG* showed positive correlations with the tumor grade of ESCC and were up‐regulated in G3 (poorly‐differentiated) compared to G1 (well‐differentiated). ARTN2, which encodes aryl hydrocarbon receptor nuclear translocator, is a transcriptional factor related to adaptive responses 29. Over‐expression of ARNT2 is responsible for the down‐regulation of HIF1‐α and leads to cell growth and proliferation in oral squamous cell carcinoma 30. ARNT2 expression is significantly lower in nonsmall cell lung cancer (NSCLC) compared to normal tissues. Inhibition of ARNT2 expression promotes NSCLC cell growth in a xenograft model 31. Beyond that, increased ARNT2 inhibits cell proliferation, invasion and metastasis in hepatocellular carcinoma (HCC) and over‐expression of ARNT2 is the independent prognostic factor of overall survival and tumor to recurrence in HCC 32. *PPARG* encodes peroxisome proliferator activated receptor γ, which is a member of PPAR subfamily of nuclear receptor. PPARG was significantly down‐regulated in ESCC tissues compared to matched mucosa tissues, for which the results were not compatible with our bioinformatics analyses showing that *PPARG* was significantly up‐regulated in ESCC with G3 (poorly‐differentiated) compared to those with G1 (well‐differentiated) and G2 (moderately‐differentiated). However, the expression status of *PPARG* in GSE23400 was in agreement with previous studies. PPARG is reported to function as an oncogene in ESCC and the activation of PPARG suppresses cell proliferation and induces cell apoptosis of esophageal cancer cells by inhibiting the TLR‐4 dependent mitogen‐activated protein kinase pathway 33.

*CDKN1A*, also known as CDKN1 and P21, encodes cyclin‐dependent kinase inhibitor 1A. It contributes to regulate cell‐cycle progression by inhibiting the activity of cyclin‐cyclin‐dependent kinase 2/4. In the present study, the expression level of *CDKN1A* was negatively correlated with the histological grade of ESCC. It was significantly down‐regulated in ESCC patients with G3 (poorly‐differentiated) compared to those patients with G1 (well‐differentiated). Decreased expression of nucleostemin suppresses cell proliferation of esophageal carcinoma by up‐regulating CDKN1A 34. Trichostatin A, a histone deacetylase inhibitor, suppresses cell proliferation and enhances cell G1 phase arrest by inducing the expression of CDKN1A and p27 35. miR‐34a functions as anti‐tumor effect by increasing the expression level of p53/CDKN1A 36.

The dysregulated genes correlated with the tumor grade of ESCC were identified by bioinformatics analyses based on the TCGA database in the present study. To access the discriminatory ability of those dysregulated genes between ESCC patients with G1 and those with G2, the counts of candidate genes originating from 188 ESCC patients incorporated into our in silicon analysis in the TCGA database were used to depict the ROC curve. The ROC analyses showed that the AUC of *CDKN1A*,* GOLGA7B* and *TIAF1* was greater than 0.85, indicating that *CDKN1A* and *GOLGA7B* might be potential biomarkers for discriminating ESCC patients with G1 (well‐differentiated) from those with G3 (poorly‐differentiated).

To detect the expression levels of candidate genes in ESCC tissues, the GSE23400 dataset was used in our analyses. The expression level of *TIAF1*,* LAMB3* and *PPARG* was statistically different between ESCC tissues and matched mucosa tissues; however, the expression levels of *GOLGA7B*,* GOLGA1*,* ARNT2* and *CDKN1A* were not statistically different between ESCC and matched mucosa tissues. There is heterogeneity of the expression status of candidate genes between the TCGA and GSE23400 datasets. Two confounding factors might contribute to the the contradictory results of the expression status of candidate genes in ESCC between the these datasets. First, different sequencing platforms were used. The mRNA expression profiling of the GSE23400 dataset was based on an Affymetrix Human Genome U133A Array and an Affymetrix Human Genome U133B Array and the mRNA expression profiling of the TCGA dataset was based on RNA‐sequencing. Different sequencing platforms might lead to heterogeneity between the TCGA and GSE23400 datasets. Second, there were different procurement countries for the ESCC samples available in the respective datasets. The procurement countries of ESCC samples in the TCGA dataset included Netherlands, USA, Russia, Ukraine and Canada, whereas the ESCC samples in the GSE23400 dataset were obtained from patients who underwent surgery in China.

The present study comprises a preliminary analysis for the identification of genes associated with the tumor grade of ESCC. There are limitations to our study. First, several candidate genes associated with the tumor grade of ESCC were identified, although the biological roles of corresponding genes with respect to cell differentiation, migration and adhesion of ESCC were not explored through *in vivo* and *in vitro* experiments. Second, the expression levels of candidate genes in ESCC patients compared to matched mucosa tissues and in ESCC patients with well‐, moderate‐ and poor differentiation need to be validated in a large sample size of ESCC patients in future studies. Third, the diagnostic value of *CDKN1A*,* GOLGA7B* and *TIAF1* needs to be validated by use of a large cohort in clinical practice.

## Conclusions

Taken together, the association between dysregulated genes and the tumor grade of ESCC was analyzed based on the metadata of patients retrieved from the TCGA database. In total, 1032 dysregulated genes associated with the tumor grade in ESCC were identified and these genes were significantly enriched in the phospholipase D signaling pathway, as well as in pathways involved in cancer and small cell lung cancer. *CDKN1A*,* GOLGA7B* and *TIAF1* have potential diagnostic value for discriminating ESCC patients with G1 (well‐differentiated) from those with G3 (poorly‐differentiated).

## Author contributions

CL designed the experiment. CL and JX analyzed the data. JX and CL collected patient information. JX and CL drafted the manuscript.

## Supporting information


**Table S1.** The full list of genes associated with the tumor grade in ESCC.

**Table S2.** The identified differentially expressed genes in G1 versus G2, G2 versus G3 and G3 versus G1.
